# Predictive value of multimodal neurological monitoring in the postoperative neurological dysfunction after cardiovascular surgery with cardiopulmonary bypass

**DOI:** 10.3389/fneur.2026.1834632

**Published:** 2026-06-04

**Authors:** Miao Zou, Xiang Tan, Xinli Zhang, Mengqiu Yi, Yan Xiang, Peng Wan

**Affiliations:** Department of Critical Care Unit, The First Clinical Medical College of Three Gorges University, Yichang, Hubei, China

**Keywords:** cardiopulmonary bypass, cardiovascular surgery, multimodal neurologic monitoring, neurologic dysfunction, sensitivity

## Abstract

**Objective:**

The use of multimodal neurologic monitoring (MNM) has rarely been reported in monitoring patients after cardiovascular surgery with cardiopulmonary bypass. This prospective observational cohort aims to the effectiveness of MNM in predicting neurologic dysfunction during the postoperative period for the patients undergoing cardiovascular surgery with cardiopulmonary bypass.

**Methods:**

A total of 156 patients who remained unawake 6 h after ICU admission (Glasgow Coma Scale [GCS] score < 8) were enrolled. These patients were monitored using a quantitative electroencephalogram (qEEG) and transcranial Doppler ultrasound. Neurologic dysfunction was classified according to the American College of Cardiology classification for neurologic injury following cardiac surgery, which includes Type I (focal injury, such as stroke diagnosed by CT/MRI, or coma assessed by GCS) and Type II (global injury, such as delirium assessed by confusion assessment method for the ICU [CAM-ICU], or cognitive dysfunction evaluated by MoCA). The patients were grouped into non-neurologic (*n* = 85) and neurologic dysfunction groups (*n* = 71).

**Results:**

The duration of extracorporeal circulation and extubation were significantly longer in the neurologic dysfunction group than the non-neurologic dysfunction group (*p* < 0.05). The abnormal amplitude-integrated electroencephalogram (aEEG), relative alpha variability (RAV) grade (III-IV), and pulsatility index (PI) of patients in the neurologic dysfunction group were higher and the band energy percentage (*α*%) and end diastolic velocities (EDVs) were lower than the non-neurologic dysfunction group (*p* < 0.05). Age, gender, days of tracheal intubation, aEEG, and RAV classification were significantly different between the subgroups that underwent different surgical procedures (*p* < 0.05). The RAV + A% + EDV + PI combination AUC for predicting neurologic dysfunction was 0.735 (95% CI: 0.658–0.812) with a specificity of 0.843 and a sensitivity of 0.507, which was better than other combined indicators (*p* < 0.001).

**Conclusion:**

MNM can monitor the 24-h changes in postoperative brain function among these targeted patients. The AUC of RAV + *α*% + EDV + PI combination for predicting neurologic dysfunction was better than other combined or single indicators. Given its relatively low sensitivity, the RAV + *α*% + EDV + PI combination is suggested to serve best as a tool for identifying high-risk patients warranting intensified monitoring and preemptive neuroprotective strategies, rather than a standalone diagnostic test.

## 1 Introduction

Postoperative neurologic dysfunction after cardiovascular surgery may lead to significant morbidity and mortality (1, 2). Brain injury is one of the main factors associated with a poor prognosis of patients after cardiovascular surgery. Reduced cerebral blood flow during extracorporeal circulation is the main cause of ischemic brain damage (1, 2). However, during cardiopulmonary bypass (CBP), a series of pathophysiologic reactions can take place, including systemic neuroinflammation, a pathologic stress response, coagulation disorders, and organ hypoperfusion. Additionally, factors like anesthesia drugs, temperature management, non-pulsating blood flow, and ischemia reperfusion also significantly impact the postoperative neurologic status (3–6). Although temperature reduction protects vital organ function, further temperature reduction does not reduce metabolic rate, but leads to cranial hypoperfusion injury and additional complications (4, 7). This effect may further worsen prognosis and lead to a poor quality of life.

Intraoperative multimodal neurologic monitoring (MNM) has been reported to result in a significant reduction of neurologic complications (8). The main MNM monitoring tools include non-invasive methods, including intracranial pressure monitoring technology, brain tissue oxygen monitoring, cerebral oxygen metabolism monitoring, electroencephalography (EEG) monitoring, cerebral blood flow monitoring, and cerebral microdialysis technology. MNM can guide precise treatment and provide timely and accurate brain function information for prognostic assessment (8, 9). Most of the previous MNM-related studies have been applied in patients with traumatic craniocerebral injury, post-cardiac arrest cardiopulmonary resuscitation, epilepsy monitoring, and sedative medication guidance. MNM is often used intraoperatively for cardiac surgeries (8, 10).

At present, there are several core gaps in research on the application of postoperative MNM in patients undergoing cardiovascular surgery combined with CPB. First, most studies focus on intraoperative monitoring of MNM, with relatively few studies on the postoperative periods. The first 24 h after ICU discharge is a critical stage for the occurrence and development of brain injury. Second, existing studies have not clearly defined the optimal combination of monitoring indicators and critical values for MNM, and mostly adopt single indicators or simple combinations that limit predictive efficacy. Lastly, targeted analyses for high-risk groups who remain unresponsive six hours post-surgery are lacking, which restricts the clinical applicability of the findings.

Therefore, this study was conducted to validate the value of MNM in predicting postoperative neurologic function in patients who remain unawake 6 h following cardiovascular surgery and CPB.

## 2 Methods

### 2.1 Patients

Patients were enrolled at our hospital from January 2023 to March 2025 in this prospective observational cohort. We included the patients receiving cardiovascular surgery followed by CPB who were at high risk of remaining unresponsive in the first six hours postoperatively. This was an exploratory, single-center study without *a priori* statistical power analysis. This study was approved by the Medical Ethics Committee of our hospital (approval number: 2023–171-01). Written informed consent was obtained from each patient or their family members.

### 2.2 Inclusion and exclusion criteria

The inclusion criteria were as follows: (i) patients eligible to undergo cardiovascular surgery followed by CPB; (ii) patients transferred to the intensive care unit (ICU) for postoperative monitoring; (iii) patients who were not awake 6 h after admission to the ICU (Glasgow coma score [GCS] < 8) (11); and (iv) patients with temporal fenestrum measurements and good audible visible blood flow signals under transcranial Doppler ultrasound (TCD) examinations. The inclusion criterion (GCS < 8 at 6 h) was intentionally chosen to enhance the study cohort with patients who were at high risk for neurologic complications, as an early failure to awaken is a recognized sign of possible brain injury following CPB. Consequently, the findings of this study are mainly relevant to this high-risk group and may not be applicable to all patients who have an uncomplicated early awakening after cardiovascular surgery.

The exclusion criteria were as follows: (i) death within 72 h after surgery due to cardiac arrest or massive bleeding; (ii) age < 18 years; (iii) TCD silent window during which a cerebral blood flow signal could not be obtained; (iv) history of neuropsychiatric disease and middle cerebral artery (MCA) stenosis; (v) did not consent to MNM; (vi) a co-existing terminal disease; (vii) incomplete data from related studies; and (viii) patients with EEG fragments when the midazolam dose was > 2 mg/h or the propofol dose was > 2 ug/(kg/min) were excluded, to minimize the inhibitory effect of high-dose sedative drugs on brain electrical activity to the greatest extent.

All patients underwent cardiovascular surgery performed by the same group of surgeons with over 10 years of experience. Upon admission to the department for intervention, brain protection measures were implemented, including glucose (80–180 mg/dL), hemoglobin (7–9 g/dL), SpO_2_ (94–97%), Na^+^ (145–155 mmol/L), avoidance core temperature > 38 °C, adequate sedation and analgesia, mean arterial pressure (≥ 80 mmHg), and pCO_2_ (35–40 mmHg). ICU care was provided by the same team of skillful nurses. The MNM on neurologic function began 6 h after sedation in the ICU. The goal of sedation in this study was to maintain sedation for 6 h after the termination of sedation, targeting a shallow sedation level with a Richmond-Agitation-Sedation Scale (RASS) score between −2 and 0, without the use of muscle relaxants. A dedicated neuro-intensive care nurse would assess the RASS score every hour using a blinded method, and adjust the drug dosage to maintain consistency in the sedation plan. All patients received a standardized sedation protocol. Dexmedetomidine (0.2–0.7 μg/kg/h) and fentanyl (0.5–1.5 μg/kg/h) were used for postoperative sedation and analgesia, titrated to a RASS score of −2 to 0. Midazolam (0.5–2 mg/h) or propofol [0.5–2 μg/(kg/min)] was added if additional sedation was required. EEG fragments obtained when midazolam >2 mg/h or propofol >2 μg/(kg/min) were excluded to minimize drug-induced EEG suppression, as these doses are considered moderate-to-high in the postoperative ICU setting.

CPB was mandatory for all procedures included in this study. CPB time was defined as the duration from cannulation to decannulation. Extracorporeal membrane oxygenation (ECMO) cases, were analyzed separately due to the physiological differences in cerebral pulsatile flow.

### 2.3 Data collection

The following clinical data of the patients were collected: (i) general information, including gender, age, admission acute physiology and chronic health evaluation II (APACHE II) score, postoperative GCS score, extracorporeal circulation time, length of ICU hospital stay (days), duration of mechanical ventilation (days), total length of hospital stay (days), type of surgery, and cerebral oxygen level; (ii) quantitative electroencephalogram (qEEG) indices, amplitude-integrated electroencephalogram (aEEG), relative alpha variability (RAV), the relative proportion of an *α* wave in the frequency band energy (α%), and spectral entropy (SE); (iii) peak systolic velocities (PSVs), end diastolic velocities (EDVs), pulsatility index (PI), and resistance index (RI) of the MCA; (iv) neurologic dysfunction grouping based on the 2004 American College of Cardiology classification of cranial nerve injury after cardiac surgery (1) and grouping based on the clinical manifestations of patients after discontinuation of sedatives; SE was determined by analyzing the stable data after the baseline automatically derived by the software.

The American College of Cardiology classified two major types of postoperative cerebral neurological injury after cardiac surgery (1): type I, which includes fatal or non-fatal strokes, coma, or movement disorders; and type II, which includes cognitive dysfunction, memory loss, seizures, or delirium. The presence of neurologic dysfunction was determined according to the 2004 American College of Cardiology typology of cranial nerve injury after cardiac surgery. Patients were then categorized into two groups: those with non-neurologic dysfunction and those with neurologic dysfunction (types I and II).

Stroke can be diagnosed by head CT and MRI. Coma is diagnosed based on the GCS score (Supplementary File). Delirium was assessed using the RASS and confusion assessment method for the ICU (CAM-ICU) scores (Supplementary File). The CAM-ICU evaluation was performed when the RASS score exceeded −4. Due to all patients being intubated, the mini-mental state examination (MMSE) was not utilized to evaluate these patients. The assessment focused on four core indicators: fluctuations in consciousness, lack of concentration, disordered thinking, and changes in consciousness level. The assessment was conducted by two physicians, ensuring the objectivity of the evaluation. To evaluate cognitive dysfunction following extubation, the study utilized the Montreal Cognitive Assessment Scale (MoCA) 24–48 h post-extubation, after the sedative drugs had fully metabolized. A MoCA score of less than 26 points was set as the diagnostic criterion for cognitive dysfunction. The diagnosis of epileptic seizures primarily relied on qEEG monitoring of epileptic wave release or visual myoclonic seizures.

The patients were analyzed in various subgroups according to the different surgical procedures performed.

### 2.4 qEEG monitoring

The international American Niko power electroencephalogram was used. The 8-lead disc electrodes were placed according to the international 10–20 system. Specifically, the electrodes were placed at the left frontal pole of the brain, the right frontal pole of the brain, the left center of the brain, and the right center of the brain with the midline point at the top of the brain as the reference electrode and the middle line point at the frontal pole of the brain as the grounding electrode. The indicators of qEEG monitoring were mainly observed, as follows: ① The aEEG trend was classified as grade I (normal amplitude, upper scale >10 μV, and lower scale >5 μV), grade II (mildly abnormal amplitude, upper scale <10 μV, and lower scale >5 μV or upper scale >10 μV and lower scale <5 μV), and grade III (moderately abnormal amplitude, upper scale <10 μV, and lower scale <5 μV). ② The RAV was quantified and compressed by an EEG and presented in the form of trend maps, which were extracted and analyzed every 2 min on average. The RAV baseline was taken as the mean value of the relative *α* percentage every 4 h. RAV was determined by calculating the percentage change in alpha band power relative to a moving baseline. Briefly, a mean alpha percentage was established over a 4-h baseline, and subsequent measurements were then compared to this baseline. RAV was then semi-quantitatively graded based on the degree of variation from the baseline: Grade I (4 points): variation >15%; Grade II (3 points): variation 10–15%; Grade III (2 points): variation 2–10%; Grade IV (1 point): variation < 2% (12). The average RAV grade during the 24-h monitoring period was used for analysis. The *α* variation score of each monitoring time period was manually recorded and the average value was used for comparison and analysis. Human interference and electrode sheet were excluded from falling off. The *δ* (0.5–4 Hz), *θ* (4–8 Hz), *α* (8–13 Hz), and *β* (13–30 Hz) were directly read as percentages. Nichole software automatically exports continuous SE values and collects the continuous SE values after baseline stabilization for analysis.

### 2.5 TCD-related metrics

Specific of the MCA PSV, EDV, PI, and RI values were obtained in the temporal window using a phased-array probe at 2–2.5 MHz. The measurements were performed at three timepoints (6, 12, and 24 h after surgery). The measurements at each time point were completed within 30 min, and the average value of three measurements was taken to ensure the reliability of the data. Six hours after the anesthetic drugs are metabolized, neuroinflammation begins at the 12-h mark, and by 24 h, there is a period of relative stability in the indicators of brain injury. Only the 24-h monitoring data were included, as no significant changes in early blood flow signals were noted. Adjustments were made to the probe position and angle to minimize artifacts during measurement. The assistant assisted the patient in maintaining a passive position to reduce motion artifacts. Additionally, the device’s gain and depth were adjusted, and the appropriate filtering was selected to ensure measurement accuracy. The measurements were conducted by the same experienced physician.

### 2.6 Neurological outcome assessment timeline and protocol

Neurological function assessments were performed using standardized tools at specific time points. The GCS was measured at 6 h upon ICU admission and then daily at 8:00 a.m. after the withdrawal of sedation. CAM-ICU was assessed twice daily (8:00 a.m. and 8:00 p.m.) from ICU admission until extubation or resolution of delirium, always preceded by RASS assessment to confirm arousability.

### 2.7 Statistical methods

SPSS 26.0 statistical software was used for data collation and analysis. Measurement information is expressed as x ± s if a normal distribution were met and two independent samples t-tests were used for comparison between groups. If a normal distribution was not met, the median (upper and lower quartiles) was used. Comparisons between groups were made using the Mann–Whitney U test. Count data are expressed as frequencies (percentages) and comparisons between groups were made using the chi-square test. The Mann–Whitney U test was used for between-group comparisons of rank information. Multifactorial analysis was performed using binary logistic regression analysis. ROC curve analysis was used for risk prediction efficacy analysis. A *p* < 0.05 was considered statistically significant.

## 3 Results

A total of 190 patients were enrolled in the current study. Six patients who died within 72 h, 8 patients < 18 years of age, 4 patients who had a previous MCA stenosis, and 16 patients with incomplete data collection were excluded based on the exclusion criteria. Therefore, 156 patients were studied, including 101 males and 55 females.

### 3.1 Comparison of general information of patients in the two groups

The patients were divided into non-neurologic (*n* = 85) and neurologic dysfunction groups (*n* = 71). The general data for both groups were comparable in terms of gender, age, admission APACHE II score, postoperative GCS score, length of ICU stay in days, total days of hospitalization, type of surgery, cerebral oxygen levels, hypothermia levels, and cerebral perfusion (*p* > 0.05; Table 1). The median extracorporeal circulation time in the neurologic dysfunction group was 143.00 (133.00, 148.00) min, which was longer than that of non-neurologic dysfunction group (137.00 [132.25, 144.00] min) (*p* = 0.035, Table 1). The median duration of tracheal intubation for the neurologic dysfunction group was 4.00 days, which was longer than that for the non-neurologic dysfunction group (2.5 days) (*p* = 0.029, Table 1).

**Table 1:** Comparison of general information of patients in the two groups.

Indicators	Non-neurologic dysfunction group (*n* = 85)	Neurologic dysfunction group (*n* = 71)	*χ* ^2^ */z*	*p*
Gender, *n* (%)			1.841	0.175
Male	51 (60.0)	50 (70.4)		
Female	34 (40.0)	21 (29.6)		
Age (y)	57.00 (49.50, 63.50)	57.00 (49.00, 66.00)	−0.538	0.591
Admission APACHE II	15.00 (10.00, 25.00)	15.00 (12.00, 23.00)	−0.877	0.380
Postoperative GCS score	10.00 (8.00, 13.00)	10.00 (9.00, 13.00)	−0.760	0.447
Extracorporeal circulation time (min)	137.00 (132.25, 144.00)	143.00 (133.00, 148.00)	−2.106	0.035*
Days in ICU (d)	4.00 (2.00, 7.00)	5.00 (4.00, 7.00)	−1.901	0.057
Days of tracheal intubation(d)	2.50 (1.00, 6.00)	4.00 (2.00, 6.25)	−2.181	0.029*
Total hospital days (d)	17.00 (11.00, 23.00)	17.00 (14.00, 21.00)	−1.420	0.156
Brain oxygen value (%)	62.00 (57.00, 65.00)	60.00 (48.00, 72.00)	−0.242	0.808
Type of surgery, *n* (%)			5.576	0.062
Sun procedure	31 (36.5)	35 (49.3)		
Coronary artery bypass grafting	36 (42.4)	30 (42.3)		
Others	18 (21.2)	6 (8.5)		
Hypothermia level			0.343	0.558
Moderate (28–32 °C)	53 (62.4)	41(57.7)		
Deep (< 28 °C)	32 (37.6)	30(42.3)		
Cerebral perfusion, *n* (%)			0.302	0.582
Antegrade	26 (76.5)	24(70.6)		
Retrograde	8 (23.5)	10(29.4)		

APACHE II, acute physiology and chronic health evaluation II; GCS, Glasgow coma scale; ICU, intensive care unit; *p < 0.05.

### 3.2 Comparison of general information of patients in the different subgroups

There were 66, 66, and 24 patients who underwent the Sun’s procedure, coronary artery bypass grafting, and other surgical procedures (traditional open thoracotomy and surgeries involving the aortic arch), respectively.

The length of stay in the ICU (days), days of tracheal intubation, GCS score, hypothermia level, aEEG, and RAV classification were significantly different among the subgroups that underwent various surgical procedures (*p* < 0.05; Table 2).

**Table 2:** Comparison of general information between types of surgery.

Indicators	Sun’s procedure	Coronary artery bypass grafting	Others	Total	*p*
Gender, *n* (%)				4.630	0.099
Female	22(34.4)	19(28.8)	13(54.2)		
Male	42(65.6)	47(71.2)	11(45.8)		
Age (y)	57.50 (52.00–62.00)	57.00 (50.00–66.00)	47.50 (30.50–67.50)	2.736	0.255
Extracorporeal circulation time (min)	140.67 ± 8.68	138.36 ± 9.89	136.71 ± 11.33	1.804	0.168
Brain oxygen value (%)	61.71 ± 12.41	61.33 ± 8.08	58.96 ± 6.75	0.691	0.503
Days in ICU (d)	7.00 (5.00–9.00)	4.00 (2.00–5.00)	3.50 (1.50–7.00)	25.854	< 0.001*
Days of tracheal intubation (d)	5.00 (2.00–7.00)	3.00 (2.00–6.00)	1.00 (1.00–3.00)	18.210	< 0.001*
GCS score at 6 h upon ICU admission	11.00 (9.00–13.00)	9.00 (7.00–12.00)	11.50 (9.00–13.50)	6.943	0.031
Total hospital days (d)	18.50 (14.00–25.00)	17.00 (13.00–23.00)	14.00 (10.50–19.50)	5.944	0.051
Undesirable complications				5.576	0.062
Non-neurologic dysfunction	31(47.0)	36(54.5)	18(75.0)		
Neurologic dysfunction	35(53.0)	30(45.5)	6(25.0)		
Hypothermia level				112.725	<0.001*
Moderate (28–32 °C)	8 (12.1)	66 (100.0)	20 (83.3)		
Deep (< 28 °C)	58 (87.9)	0 (0)^a^	4 (16.7)^ab^		
Cerebral perfusion, *n* (%)				-	0.169
Antegrade	49 (75.4)		1 (33.3)		
Retrograde	16 (24.6)		2 (66.7)		
aEEG, *n* (%)				7.060	0.029*
Normal	32(48.5)	40(60.6)	19(79.2)		
Abnormal (moderate, severe)	34(51.5)	26(39.4)	5(20.8)		
α (%)	3.00 (3.00–4.00)	3.00 (2.00–4.00)	3.00 (2.00–4.00)	1.555	0.460
β (%)	3.00 (2.00–4.00)	3.00 (2.00–4.00)	3.00 (2.00–4.00)	0.715	0.700
δ (%)	10.00 (9.00–13.00)	10.00 (9.00–12.00)	11.00 (9.00–12.00)	1.107	0.575
θ (%)	83.00 (80.00–85.00)	83.00 (81.00–85.00)	83.00 (80.00–86.00)	1.755	0.416
RAV classification				6.583	0.037*
Grade I–II	28 (42.4)	38 (57.6)	17 (70.8)		
Grade III–IV	38 (57.6)	28 (42.4)	7 (29.2)		
SE (%)	52.50 (49.00–57.00)	53.00 (51.00–57.00)	52.00 (49.00–56.00)	1.853	0.396
PSV	81.53 (79.00–88.00)	81.40 (79.00–88.00)	82.00 (79.75–91.70)	1.303	0.521
EDV	32.58 (29.00–36.00)	32.29 (29.00–36.00)	31.98 (27.30–36.70)	1.11	0.57
PI	1.27 (1.19–1.33)	1.26 (1.19–1.36)	1.26 (1.15–1.34)	0.632	0.729
RI	0.68 (0.59–0.71)	0.68 (0.62–0.72)	0.67 (0.59–0.71)	0.1818	0.913

^a^Indicates the use of two independent samples t-test.

^b^Indicates the use of Mann-Whitney U test.

*p < 0.05.

A univariate multimodal index analysis was performed to provide early warning of neurologic dysfunction across four subgroups, including moderate-to-deep hypothermia and retrograde perfusion, according to the intraoperative strategies outlined in the subgroup analysis (Tables 3–6).

**Table 3:** Comparison between the patients with moderate hypothermia in the two groups.

Indicators	Non-neurologic dysfunction group with moderate hypothermia (*n* = 53)	Neurologic dysfunction group with moderate hypothermia (*n* = 41)	*χ* ^2^ */z*	*p*
aEEG, *n* (%)			1.384	0.239
Normal	36 (67.9)	23 (56.1)		
Abnormal (moderate, severe)	17 (32.1)	18 (43.9)		
α (%)	4.00 (3.00, 4.00)	3.00 (2.00, 3.00)	−2.648	0.008
β (%)	3.00 (2.00, 4.00)	3.00 (2.00, 3.00)	−1.043	0.297
δ (%)	11.00 (9.00, 12.00)	10.00 (9.00, 12.50)	−0.532	0.595
θ (%)	83.00 (80.00, 84.00)	84.00 (81.00, 86.00)	−2.045	0.051
RAV classification			1.484	0.223
Grade I–II	35 (66.0)	22 (53.7)		
Grade III–IV	18 (34.0)	19 (46.3)		
SE (%)	52.00 (50.00, 57.00)	55.00 (49.50, 57.00)	−0.136	0.892
PSV	82.00 (79.00, 86.85)	80.00 (79.00, 88.55)	−0.987	0.324
EDV	33.59 ± 2.68	32.08 ± 3.22	2.477	0.015
PI	1.21 (1.14, 1.34)	1.27 (1.20, 1.35)	−1.932	0.053
RI	0.67 (0.52, 0.70)	0.69 (0.63, 0.75)	−2.552	0.011

**Table 4:** Comparison between the patients with deep hypothermia in the two groups.

Indicators	Non-neurologic dysfunction with deep hypothermia (*n* = 32)	Neurologic dysfunction group with deep hypothermia (*n* = 30)	*χ* ^2^ */z*	*p*
aEEG, *n* (%)			10.872	0.001
Normal	23 (71.9)	9 (30.0)		
Abnormal (moderate, severe)	9 (28.1)	21 (70.0)		
α (%)	3.00 (2.00,4.75)	3.00 (2.00,4.00)	−0.558	0.577
β (%)	3.00 (2.00,4.00)	3.00 (2.00,4.00)	−0.118	0.906
δ (%)	10.00 (9.00,12.75)	12.00 (8.75,14.00)	−1.381	0.167
θ (%)	84.00 (81.25,85.75)	83.00 (79.75,85.25)	−1.034	0.301
RAV classification			5.565	0.018
Grade I–II	18 (56.3)	8 (26.7)		
Grade III–IV	14 (43.8)	22 (73.3)		
SE (%)	55.00 (50.00,59.00)	52.00 (49.00,56.00)	−1.414	0.157
PSV	83.25 (79.15,88.00)	81.00 (79.00,88.93)	−0.586	0.558
EDV	34.01 ± 2.81	32.47 ± 3.42	1.947	0.056
PI	1.22 (1.16,1.29)	1.32 (1.21,1.39)	−2.842	0.004
RI	0.67 (0.63,0.69)	0.65 (0.54,0.72)	−0.346	0.730

**Table 5:** Comparison of the patients with antegrade cerebral perfusion in the two groups.

Indicators	Non-neurologic dysfunction group with antegrade cerebral perfusion (*n* = 26)	Neurologic dysfunction group with antegrade cerebral perfusion (*n* = 24)	*χ* ^2^ */z*	*p*
aEEG, *n* (%)			11.538	0.001
Normal	19 (73.1)	6 (25.0)		
Abnormal (moderate, severe)	7 (26.9)	18 (75.0)		
α (%)	3.00 (2.75,4.25)	3.00 (2.00,4.00)	−0.941	0.347
β (%)	3.00 (2.00,4.00)	3.00 (2.00,3.00)	−0.765	0.444
δ (%)	10.00 (9.00,13.00)	11.00 (9.00,14.00)	−0.324	0.746
θ (%)	83.00 (80.75,85.25)	83.00 (80.00,86.75)	−0.156	0.876
RAV classification			3.978	0.046
Grade I–II	16 (61.5)	8 (33.3)		
Grade III–IV	10 (38.5)	16 (66.7)		
SE (%)	55.50 (50.75,59.00)	51.00 (49.00,54.50)	−2.224	0.026
PSV	82.75 (79.00,87.63)	80.80 (79.00,87.70)	−0.613	0.540
EDV	34.36 ± 2.74	31.69 ± 2.80	3.401	0.001
PI	1.23 (1.14,1.29)	1.30 (1.21,1.40)	−2.783	0.005
RI	0.67 (0.63,0.71)	0.69 (0.60,0.77)	−1.099	0.272

**Table 6:** Comparison of the patients with retrograde cerebral perfusion of patients in the two groups.

Indicators	Non-neurologic dysfunction group with retrograde cerebral perfusion (*n* = 8)	Neurologic dysfunction group with retrograde cerebral perfusion (*n* = 10)	*χ* ^2^ */z*	*p*
aEEG, *n* (%)	-	0.637		
Normal	5 (62.5)	4 (40.0)		
Abnormal (moderate, severe)	3 (37.5)	6 (60.0)		
α (%)	3.50 (2.00, 5.00)	3.00 (2.75, 4.00)	−0.501	0.617
β (%)	3.00 (3.00, 3.75)	3.50 (3.00, 4.25)	−1.057	0.290
δ (%)	10.00 (8.25, 12.75)	13.00 (9.50, 14.00)	−1.123	0.261
θ (%)	83.50 (81.25, 84.75)	80.50 (79.00, 84.25)	−0.982	0.326
RAV classification			-	1.000
Grade I–II	3 (37.5)	3 (30.0)		
Grade III–IV	5 (62.5)	7 (70.0)		
SE (%)	53.50 (47.25, 60.00)	53.50 (50.50, 56.25)	−0.178	0.859
PSV	84.00 (81.00, 90.25)	81.00 (78.75, 90.35)	−0.671	0.503
EDV	32.81 ± 2.68	34.63 ± 3.53	−1.204	0.246
PI	1.21 (1.17, 1.28)	1.29 (1.19, 1.38)	−0.933	0.351
RI	0.68 (0.57, 0.69)	0.53 (0.51, 0.61)	−2.144	0.032

### 3.3 Univariate analysis of early warning indicators of neurologic dysfunction

Two independent samples t-tests, the Mann–Whitney U-test, and the chi-square test showed no significant differences in *β*-relative band energy, *θ*-relative band energy, *δ*-relative band energy, SE, PSV, and RI between the 2 groups (*p* > 0.05; Table 2). aEEG (*p* = 0.002) and *α*% (*p* = 0.017) were significantly more abnormal in the neurologic dysfunction group than the non-neurologic dysfunction group (Table 7). More patients exhibited grade III–IV RAV in the neurologic dysfunction group than the non-neurological dysfunction group (*p* = 0.013). The EDV was slower (*p* = 0.02) and the PI was higher (*p* = 0.001) in the neurologic dysfunction group than in the non-neurologic dysfunction group (Table 7).

**Table 7:** Univariate analyses predicting neurologic dysfunction.

Indicators	Non-neurologic dysfunction group (*n* = 85)	Neurologic dysfunction group (*n* = 71)	*χ* ^2^ */t/z*	*p*
aEEG, *n* (%)			9.431^c^	0.002*
Normal	59 (69.4)	32 (45.1)		
Abnormal (moderate, severe)	26 (30.6)	39 (54.9)		
α (%)	4.00 (2.00, 4.00)	3.00 (2.00, 4.00)	−2.379^b^	0.017*
β (%)	3.00 (2.00, 4.00)	3.00 (2.00, 4.00)	−0.723^b^	0.470
θ (%)	10.00 (9.00, 12.00)	11.00 (9.00, 13.00)	−0.611^b^	0.541
δ (%)	83.00 (81.00, 85.00)	83.00 (80.00, 86.00)	−0.769^b^	0.442
RAV classification, *n* (%)			−2.497^b^	0.013*
Grade I–II	62 (72.9)	30 (42.3)		
Grade III–IV	23 (27.1)	41 (57.7)		
SE (%)	52.00 (50.00, 58.00)	53.00 (49.00, 57.00)	−0.802^b^	0.423
PSV (cm/s)	82.00 (79.00, 87.75)	80.60 (79.00, 88.60)	−1.090^b^	0.276
EDV (cm/s)	33.75 ± 2.72	32.24 ± 3.29	3.126^a^	0.002*
PI	1.22 (1.15, 1.29)	1.29 (1.21, 1.38)	−3.222^b^	0.001*
RI	0.67 (0.62, 0.69)	0.68 (0.59, 0.73)	−1.846^b^	0.065

a Indicates two independent samples t-test.

b Indicates Mann–Whitney U-test.

c Indicates chi-square test.

*p < 0.05. aEEG, amplitude-integrated electroencephalogram; RAV: relative alpha variability; SE: spectral entropy; PSVs: peak systolic velocities; EDV: end diastolic velocity; PI: pulsatility index; RI: resistance index.

### 3.4 Determination of the optimal cut-off value for the PI

**Figure 1:**
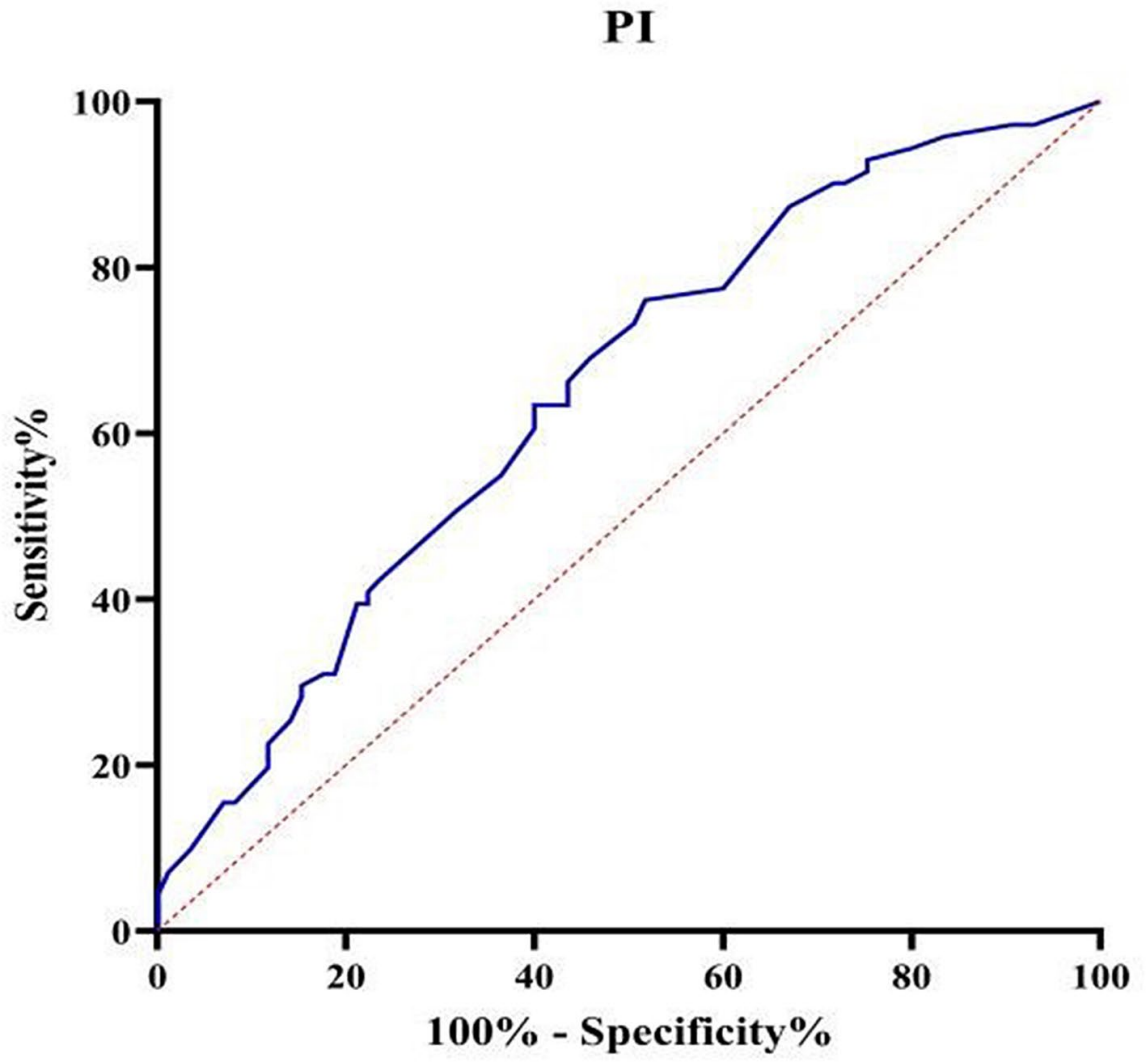
ROC curve analysis of PI for predicting neurologic dysfunction.

The PI was subjected to ROC curve analysis. The AUC of the PI for predicting neurologic dysfunction was 0.650 (0.564–0.736; *p* = 0.001), the specificity was 0.482, the sensitivity was 0.761, the Youden index was 0.243, and the optimal cut-off value was 1.205 (Table 8; Figure 1).

**Table 8:** Determination of the optimal cut-off value for the PI.

Indicators	Cut-off value	AUC	95% CI		*p* value	Specificity	Sensitivity	Youden index
			Lower	Upper				
PI	1.205	0.650	0.564	0.736	0.001	0.482	0.761	0.243

### 3.5 Binary logistic regression analysis of early warning indicators of neurologic dysfunction

Indicators with significant differences in the univariate analysis were subjected to binary logistic regression analysis. After adjusting for potential confounders such as extracorporeal circulation time and type of surgery, the MNM parameters (*α*%, grade III RAV, EDV, and PI ≥ 1.205) remained significant predictors of neurologic dysfunction (*p* < 0.05; Table 9). After excluding non-CPB procedures, the combination of RAV + *α*% + EDV + PI yielded an AUC = 0.735 (95% CI: 0.658–0.812).

**Table 9:** Multifactorial regression analysis table for predicting neurologic dysfunction.

Indicators	*β*	SE	Wald χ^2^	*p*	OR	95% CI	
						Lower	Upper
aEEG							
Normal	Refer						
Abnormal	0.430	0.398	1.168	0.280	1.537	0.705	3.351
α (%)	−0.398	0.173	5.322	0.021*	0.671	0.479	0.942
RAV classification							
Grade I–II	Refer						
Grade III–IV	0.936	0.396	5.597	0.018*	2.551	1.174	5.541
EDV	−0.167	0.065	6.717	0.010*	0.846	0.745	0.960
PI							
< 1.205	Refer						
≥ 1.205	1.201	0.419	8.215	0.004*	3.322	1.462	7.551
Type of surgery, *n* (%)							
Others	Refer						
Sun’s Procedure	1.879	0.899	4.366	0.037*	6.544	1.123	38.122
CABG	0.689	0.614	1.258	0.262	1.991	0.598	6.635
Extracorporeal circulation time (min)	0.036	0.021	2.951	0.086	1.037	0.995	1.081
Hypothermia level							
Moderate (28–32 °C)	Refer						
Deep (< 28 °C)	−1.277	0.844	2.291	0.130	0.279	0.053	1.457
Constant	−0.442	3.528	0.016	0.900	0.643		

*p < 0.05. aEEG, amplitude-integrated electroencephalogram; RAV: relative alpha variability; EDVs: end diastolic velocities; PI: pulsatility index.

### 3.6 Predictive efficacy analysis of early warning indicators of neurologic dysfunction

**Figure 2:**
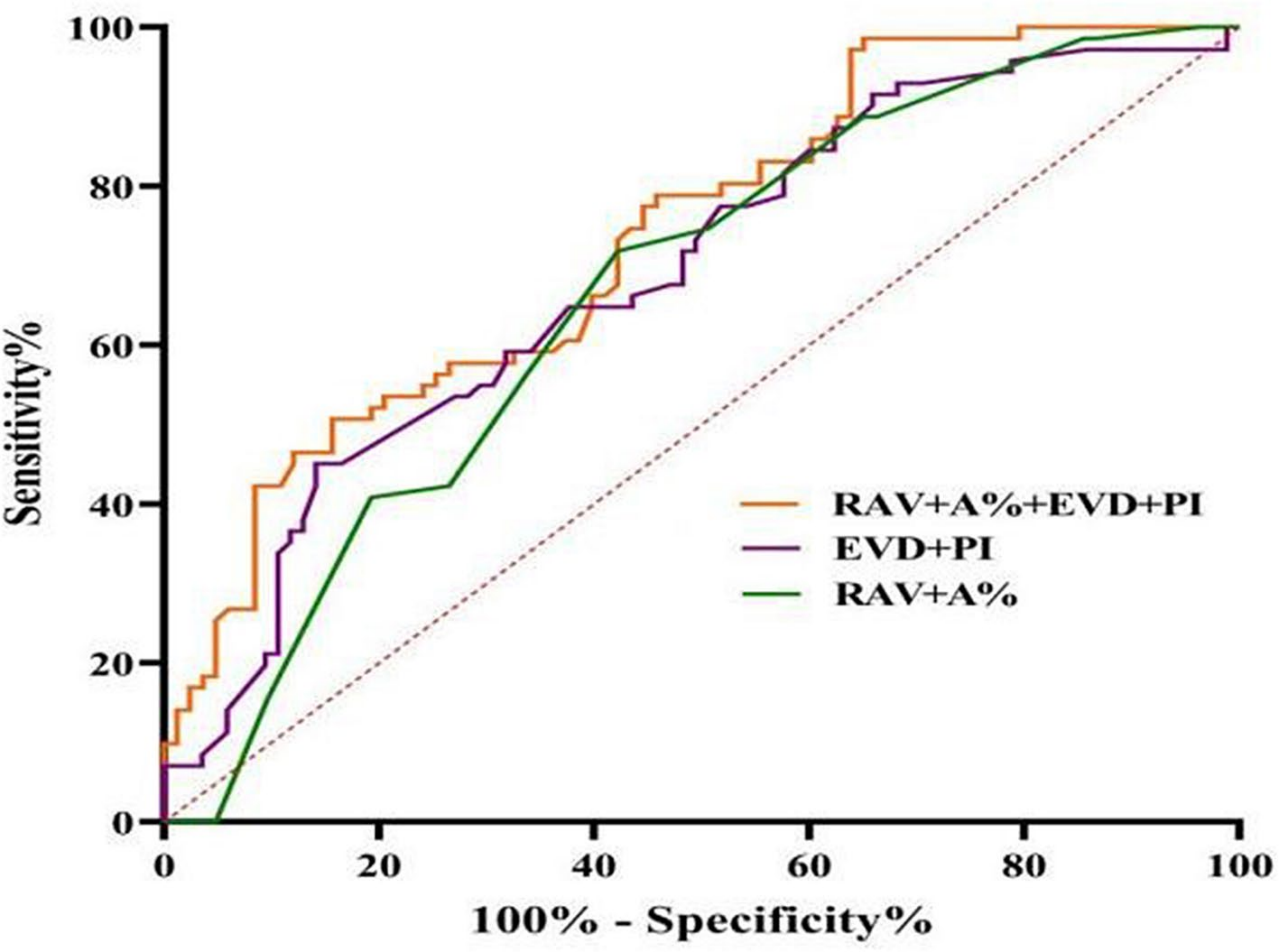
ROC curve analysis of different combination indicators for predicting the presence or absence of neurologic dysfunction.

Based on the independent predictors identified in the multivariable logistic regression model (*α*%, RAV grade III-IV, EDV, and PI ≥ 1.205), a combined predictor was generated and its predictive performance was assessed via ROC curve analysis. The α%, RAV grade, PI, and RI were subjected to ROC curve analysis for predicting the presence or absence of neurologic dysfunction. The AUC for the combined RAV + α% + EDV + PI prediction was 0.735 (0.658, 0.812; *p* < 0.001), the specificity was 0.843, the sensitivity was 0.507, and the Youden index was 0.350. The AUC for the combined EDV + PI prediction was 0.684 (0.601, 0.768; *p* < 0.001), the specificity was 0.855, the sensitivity was 0.451, and the Youden index was 0.306. The AUC for the combined RAV + α% prediction was 0.665 (0.580, 0.751; *p* < 0.001), with a specificity of 0.578, a sensitivity of 0.718, and a Jordon’s index of 0.296 (Table 10; Figure 2).

**Table 10:** Analysis of predictive efficacy of early warning indicators of neurologic dysfunction.

Indicators	*AUC*	95% CI		*P*	Specificity	Sensitivity	Youden index
		Lower	Upper				
RAV + Aα% + EDV + PI	0.735	0.658	0.812	<0.001	0.843	0.507	0.350
EDV + PI	0.684	0.601	0.768	<0.001	0.855	0.451	0.306
RAV + α%	0.665	0.580	0.751	<0.001	0.578	0.718	0.296

RAV: relative alpha variability; EDV: end diastolic velocity; PI: pulsatility index; AUC: Area Under the Curve; CI: Confidence Interval‌.

We have now analyzed the false-negative cases (*n* = 35) and found: 0 Type I (stroke/coma)35 Type II (delirium or cognitive dysfunction), of which: 22 (62.9%) had subsyndromal delirium (CAM-ICU negative but clinical features), 13 (37.1%) had mild delirium (CAM-ICU positive, short duration <48 h).

### 3.7 Subgroup analysis of type I and type II neurological injuries

The subgroup analysis of the differences in pathophysiology and severity between type I and type II injuries was shown in Table 11. The respective data for type I (*n* = 16, incidence rate 10.3%) and type II (*n* = 55, incidence rate 35.3%) demonstrated the predictive value of MNM for various types of neurological injury and also reflected the clinical differences between the two types. There were no statistically significant differences (*p* > 0.05) in gender, age, APACHE II score, CPB duration, ICU stay duration, tracheal intubation duration, total hospital stay duration, cerebral oxygenation values, aEEG, relative band energy of *α* wave, relative band energy of *β* wave, relative band energy of *θ* wave, relative band energy of *δ* wave, RAV grade, SE, PSV, EDV and RI between patients with type I and type II neurological dysfunction. However, the PI value in patients with type I neurological dysfunction was significantly higher than that in patients with type II neurological dysfunction, with a statistically significant difference (*p* < 0.05).

**Table 11:** Comparison of general information of type I and type II neurological injuries.

Indicators	Types I (*n* = 16)	Types II (*n* = 55)	*χ*^2^/*t/z*	*p*
Gender, *n* (%)			1.930^c^	0.165
Male	14 (87.5)	36 (65.5)		
Female	2 (12.5)	19 (34.5)		
Age (y)	60.69 ± 9.52	55.27 ± 10.88	1.798^a^	0.077
Admission APACHE II	20.00 (13.25, 25.75)	15.00 (11.00, 22.00)	−1.027^b^	0.304
Extracorporeal circulation time (min)	144.50 (135.25, 145.75)	140.00 (132.00, 150.00)	−0.641^b^	0.521
Days in ICU (d)	5.50 (4.00, 9.75)	5.00 (4.00, 7.00)	−0.639^b^	0.523
Days of tracheal intubation (d)	5.00 (3.00, 7.00)	4.00 (2.00, 6.00)	−0.743^b^	0.458
Total hospital days (d)	18.00 (13.00, 20.75)	17.00 (15.00, 24.00)	−0.290^b^	0.772
Brain oxygen value (%)	65.19 ± 12.99	59.80 ± 13.68	1.402^a^	0.165
aEEG, *n* (%)			0.015^c^	0.904
Normal	7 (43.8)	25 (45.5)		
Abnormal (moderate, severe)	9 (56.3)	30 (54.5)		
α (%)	3.00 (3.00, 4.00)	3.00 (2.00, 3.00)	−1.694^b^	0.090
β (%)	3.00 (2.25, 3.00)	3.00 (2.00, 4.00)	−0.647^b^	0.518
θ (%)	10.00 (9.00, 12.00)	11.00 (9.00, 13.00)	−0.638^b^	0.523
δ (%)	83.50 (81.00, 85.00)	83.00 (80.00, 86.00)	−0.118^b^	0.906
RAV classification, *n* (%)			−0.434^b^	0.664
Grade I–II	6 (37.5)	24 (43.6)		
Grade III–IV	10 (62.5)	31 (56.4)		
SE (%)	52.00 (49.25, 57.00)	53.00 (49.00, 56.00)	−0.055^b^	0.956
PSV (cm/s)	80.50 (79.00, 88.70)	80.60 (79.00, 88.60)	−0.574^b^	0.566
EDV (cm/s)	33.41 ± 2.40	31.91 ± 3.45	1.625^a^	0.109
PI	1.34 ± 0.08	1.27 ± 0.10	2.402^a^	0.019*
RI	0.65 ± 0.09	0.67 ± 0.09	−0.584^a^	0.561

APACHE II, acute physiology and chronic health evaluation II; GCS, Glasgow coma scale; ICU, intensive care unit; RAV: relative alpha variability; EDV: end diastolic velocity; PI: pulsatility index.

^a^Indicates the use of two independent samples t-test.

^b^Indicates the use of Mann-Whitney U test.

^c^Indicates the use of the corrected chi-square test.

*p < 0.05.

## 4 Discussion

This study confirmed that MNM reflects the degree of brain injury after ischemia and hypoxia in patients undergoing cardiovascular surgery. The length of ICU stay in days, days of tracheal intubation, GCS score, hypothermia level, aEEG, and RAV classification were significantly different between the subgroups that underwent different surgical procedures. The *α*%, grade III RAV classification, EDV, and PI ≥ 1.205 under MNM may help predict neurologic-related complications postoperatively at an early stage. The RAV + α% + EDV + PI combination prediction AUC may have better application value than other combined or single indicators.

Multiple methods, such as refinement of surgery, reduction of extracorporeal circulation time, stopping the circulation at low and medium temperatures, and performing MNM, to reduce the incidence of adverse neurologic complications among cardiovascular surgery patients, have been used during the perioperative period (11, 13–16). It has been suggested that temperature control, monitoring means, and the cerebral protection strategy differ among individuals undergoing cardiac surgeries (11, 14–16). Individualized brain protection strategy should be applied based on comprehensive monitoring to balance the risks contributing to brain injury (14, 16). CPB and ECMO are two frequently used therapeutic devices among the cerebral perfusion strategies for cardiac surgery (17). CPB requires integration with the operating room table, has thicker extracorporeal circulation tubing, causes relatively greater blood damage, and requires anticoagulant drugs, such as heparin, to prevent coagulation. In contrast, ECMO tubing is designed to minimize blood injury with relatively flexible anticoagulation management (17). ECMO can substitute for cardiac output, while providing oxygenation and decarburization, although the current study did not involve patients undergoing ECMO. The VA-ECMO catheter can be inserted into the ascending aorta during thoracotomy to generate antegrade blood flow, serving as a bridge to recovery. The present study confirmed that performing MNM postoperatively is an important indicator of brain function in patients undergoing cardiovascular surgery. Patients with neurologic dysfunction typically need more time on extracorporeal circulation and extended tracheal intubation. While the monitoring dimensions and endpoint indicators in these previous studies differ from those in this research, they all highlight a common conclusion: single-modal monitoring has its limitations, whereas multi-modal combined monitoring can more comprehensively and accurately identify high-risk patients.

The incidence of neurologic dysfunction in this study was 45.5% (71/156), including both type I and II neurologic dysfunction. The incidence of postoperative stroke (type I) ranges from 1.5 to 5%, and the incidence of postoperative delirium (type II) after cardiac surgery ranges from 30 to 50% (11, 18). The current study found that the incidence rate of Type I (*n* = 16) was 10.3% while the incidence rate of Type II (*n* = 55) was 35.3%. The incidence rate of Type I was higher than that in previous literature (11, 18). This difference may be attributed to the fact that the population included in this study consisted of high-risk individuals with a GCS score of less than 8 within 6 h after surgery. Consequently, the higher incidence rate of neurological damage in this study, compared to that of the typical cardiovascular surgery patients, was understandable. In addition, we fully acknowledge that the significant differences in sample size between Type I and Type II groups may introduce heterogeneity and potential bias in the results.

Age is a risk factor for cardiovascular disease (19) but the different surgical methods used in the current study did not identify an association with age. However, another study showed that female gender was associated with long-term mortality after cardiac surgery (20). The impact of gender on postoperative outcome warrants additional investigation. A long interval to extubation mainly exists in patients after aortic dissection, which is due to the high risk of dissection (17). Longer weaning and extubation time corresponds to longer ICU lengths of stay. The reason for the statistical difference in aEEG and *β* (%) between the groups was that the cerebral blood supply is pulsed under normal cardiac pulsation, while the cerebral blood supply is flat flow during CPB (21). The cerebral blood supply under advection is worse than that under pulse and the cerebral blood supply is affected to a greater degree with the extension of CPB time. There was no significant difference in temperature and perfusion methods between the entire population in the neurologic dysfunction group, which is likely due to the small sample size in the current study. The incidence of neurologic dysfunction with deep hypothermia in aortic dissection is higher depending on the type of surgery performed. Although CPB is a common feature, the hypothermic circulatory arrest strategies in different surgeries significantly affect neurologic outcomes (4). Specifically, the Sun procedure (deep hypothermia) had higher neurologic dysfunction compared to cardiopulmonary bypass grafting.

TCD serves as an intraoperative tool to diagnose perfusion disturbances based on real-time changes in blood flow signal wave forms (11, 15). It allows for monitoring the cerebral blood flow spectra of both hemispheres, helping to assess whether bilateral cerebral perfusion is symmetric. Additionally, it enables dynamic retesting of cerebral perfusion in the same hemisphere at different times (11, 15, 16, 22). TCD measurements of the middle cerebral artery were performed at 6, 12, and 24 h after ICU admission. For the primary analysis, we focused exclusively on the 24-h data. This decision was based on two main considerations: first, the 6- and 12-h time points may be influenced by the lingering effects of intraoperative anesthetics and the dynamic stabilization of systemic hemodynamics upon ICU arrival. Second, the 24-h mark is widely recognized as a critical period when secondary brain injury processes, such as the peak of the neuroinflammatory response and the development of cerebral edema, become more pronounced. In the present study the cerebral blood flow data monitored within 24 h of admission to the Department showed that the PI was higher in the neurologic dysfunction group than the non-neurologic dysfunction group, whereas the EDV was lower than the non-neurologic dysfunction group. The subgroup analysis also suggested PI value in patients with type I neurological dysfunction was significantly higher than that in patients with type II neurological dysfunction. These findings confirm the effectiveness of TCD monitoring for assessing neurologic dysfunction. However, we recognize that omitting earlier time points may result in the loss of valuable insights into the progression of cerebral hemodynamic changes.

qEEG provides real-time, non-invasive, and reliable assessment of cerebral function as an innovative approach (9). qEEG has been reported to be effective in monitoring brain function in patients with type A aortic dissection (6, 23, 24). The results of qEEG data in the current study showed that an abnormal aEEG and RAV grading (grade III–IV) were higher in the neurologic dysfunction group than the non-neurologic dysfunction group and *α*% was lower than the non-neurologic dysfunction group. The findings confirmed that RAV grading and α% under qEEG monitoring are good predictors for early warning of neurologic dysfunction. However, qEEG is greatly affected by sedative drugs. Benzodiazepines, propofol, dexmedetomidine, and other sedative drugs can a decrease in α activity, an increase in *β* activity, and high amplitude delta waves during deep sedation. SE can also vary greatly when pain is elicited, such as occurs during bedside sputum aspiration and turning over in bed, or when the depth of sedation is different. The goal of sedation in this study was “to continue sedation for 6 h after the termination of sedation, and maintain the sedation target at the shallow sedation level of RASS score -2 to 0, and no muscle relaxants were used in all patients.” EEG fragments with a midazolam dosage exceeding 2 mg/h or propofol dosage exceeding 2 ug/(kg/min) were excluded during bedside monitoring to minimize the influence of sedative drugs on the study results. However, sedation may have some confounding factors on the results of bedside examinations but this is rarely excluded because sedation and analgesia is an important part of ICU treatment (25).

Compared with previous studies, this research presents consistent findings in multiple aspects. At the qEEG/aEEG level, Khalifa et al. (26) found that the intraoperative alpha power of patients with post-cardiac surgery delirium was significantly reduced. Guo et al. (27) confirmed that confirmed that the ROC AUC of the peak value of F3-P3 derivation index in qEEG for predicting delirium reached 0.81. In this study, the RAV classification (III-IV grades) and the significant association between *α*% and neurological dysfunction were highly consistent with those of previous research. In the TCD field, cohort studies on heart valve surgery and aortic arch surgery (28, 29) have both confirmed that low average blood flow velocity and high PI are independently associated with postoperative delirium and neurological dysfunction, which is exactly consistent with the findings in this study of elevated PI and decreased EDV.

No single monitor is adequate and perfect for neurocritical care monitoring, although the current trend involves the use of MNM (24, 25). In this study, the AUC of the RAV + α% + EDV + PI combination was 0.735 (95% CI: 0.658–0.812), with a high specificity of 0.843 and a relatively low sensitivity of 0.507. From the perspective of early warning for high-risk patients in neurocritical care, this efficacy has clear clinical value. The high specificity of 84.3% is particularly beneficial as it effectively identifies patients who are unlikely to develop neurologic dysfunction. This helps reduce false-positive alerts and prevents unnecessary escalation of care in a vulnerable postoperative group. However, the lower sensitivity of 50.7% indicates that nearly half of the patients who later developed neurologic dysfunction—mainly those with milder Type II injuries, such as subsyndromal delirium-did not show significantly abnormal MNM parameters within the first 24 h. We acknowledge this limitation clearly. The false positive rate was 15.7%, which was mainly due to abnormal MNM indicators in some patients being caused by sedative drugs or transient cerebral perfusion fluctuations. After these patients received enhanced brain protection treatment (such as optimizing blood pressure and maintaining cerebral perfusion), none of them developed clinical neurological dysfunction, suggesting that false positive results can serve as a warning signal for clinical intervention rather than a mere monitoring error. Among the false-negative cases (*n* = 35), all were Type II injuries, with the majority (62.9%) being subsyndromal delirium. These patients had shorter ICU stays and lower symptom burden. Therefore, we hypothesize that false negatives mainly occur in mild Type II injuries, although this finding should be regarded as exploratory and hypothesis-generating rather than definitive. One significant difference between this study and previous literature lies in the predictive efficacy. The AUC of the combined model of RAV + *α*% + EDV + PI in this study was 0.735, which was higher than each individual indicator but lower than the efficacy of some simple qEEG prediction models or multimodal prediction models combining cerebral oxygen and deep anesthesia status (0.81–0.903). The possible reasons for this difference include differences in patient inclusion criteria (this study limited patients with GCS < 8 who were at high risk of coma/alteration of consciousness, while most previous studies included patients after routine cardiac surgery with different baseline severity), different methods for extracting qEEG parameters. This study used RAV classification, while some studies used peak voltage, nonlinear complexity or α power spectral density, and differences in the range of definitions of neurological dysfunction (this study used the ACCA classification to cover type I/II injuries, while different studies defined the endpoints differently). These differences suggest that when selecting qEEG/TCD monitoring indicators and constructing prediction models, it is necessary to consider the baseline risk of the specific patient group and the expected incidence of neurological dysfunction individually.

Therefore, our model serves best as an auxiliary tool for identifying high-risk patients who may benefit from increased monitoring and proactive neuroprotective strategies, rather than a standalone diagnostic test. Clinicians should integrate these MNM findings with bedside clinical assessments to ensure comprehensive decision-making.

The current study had some limitations. First, the monitoring results were limited to 24 h after admission and the lack of dynamic changes caused bias. The analysis of TCD parameters was restricted to the 24-h time point. This approach, while intended to minimize early confounders, may overlook important early hemodynamic alterations. Future studies should explore the predictive value of the temporal trends in TCD variables over the first 24–72 h. Second, the sample size from a single-center study was small. The sample size was not determined by a formal power calculation but was based on a convenience sample from a single center over a defined period. Consequently, the study may be underpowered to detect smaller effect sizes for some associations, and the findings, particularly the negative results from subgroup analyses, should be interpreted with caution. Third, the type of surgery was also an independent risk factor for neurologic dysfunction. There are still many differences in CPB management for different surgical types. Fourth, the residual sedative effect of the anesthetic may also have introduced some bias though we have implemented a standardized sedation protocol and have excluded cases of excessive sedation. Fifth, although subgroup analyses of temperature and perfusion methods have been performed, more detailed analyses are needed. Sixth, only brain protection measures were applied on admission and subsequent changes in cerebral perfusion status may not be dynamically tracked in every patient, so interventions vary. Seven, the enrollment criterion of GCS < 8 at 6 h postoperatively introduces an inherent selection bias, restricting our cohort to a high-risk subgroup. This limits the generalizability of our findings to the broader, lower-risk CMVS population who awaken promptly after surgery. Future studies are warranted to evaluate the prognostic utility of MNM across the full spectrum of postoperative neurologic risk.

In the future we need to explore the clinical value of MMM-related indices in various periods, types of surgeries, and different interventions for cardiovascular surgery in a long-term, large-scale study. Subsequent studies will further collect pharmacokinetic data to explore the correlations between drug dosage, blood drug concentration, MNM indicators and neurological dysfunction, and conduct a more in-depth analysis of the drug’s effects. For the monitoring of delayed neurological complications after 24 h, based on our clinical experience in neurocritical care, a combined strategy of “early MNM + later simple neurological function monitoring” can be adopted: within 24 h after surgery, early risk stratification is completed through MNM. For high-risk patients with abnormal MNM indicators, bedside bispectral index (BIS) + daily neurological function assessment (GCS + CAM-ICU) is used for continuous monitoring from 24 to 72 h after surgery. After 7 days, MoCA is used to assess cognitive function, achieving the connection between early warning and long-term monitoring.

## 5 Conclusion

In conclusion, MNM can monitor the dynamic changes of brain function and predict the brain injury postoperatively in patients undergoing cardiovascular surgery. The *α*%, grade III RAV classification, EDV, and PI ≥ 1.205 under MNM may help predict neurologic-related complications postoperatively at an early stage. While the RAV + α% + EDV + PI combination prediction AUC outperformed other combined or single indicators, it is important to note its relatively low sensitivity. The RAV + α% + EDV + PI combination may be most effective as a supplementary tool for identifying high-risk patients who require closer monitoring and proactive neuroprotective strategies, rather than a standalone diagnostic test. Clinicians should incorporate these MNM findings with bedside clinical assessments to ensure comprehensive decision-making. What’s the most important, the overall AUC, sensitivity and specificity may mainly reflect the predictive performance for Type II outcomes, given that Type II events constituted the majority of neurological dysfunction cases.

## Data Availability

The original contributions presented in the study are included in the article/Supplementary material, further inquiries can be directed to the corresponding author.
