# Supplementary material for: Predictive value of multimodal neurological monitoring in the postoperative neurological dysfunction after cardiovascular surgery with cardiopulmonary bypass
Source: Front Neurol. 2026 Jun 4;17:1834632. doi: 10.3389/fneur.2026.1834632 (PMC13276368; doi:10.3389/fneur.2026.1834632)

**Glasgow coma scale**

| Best motor response | Best verbal respose | Eye opening |
| --- | --- | --- |
| 6 Obeys commands | 5 Oriented | 4 Spontaneous |
| 5 Localizes pain | 4 Converses but confused | 3 To verbal stimuli,command,speech |
| 4 Withdrawal from pain | 3 Inappropriate words | 2 To pain |
| 3 Flexion to pain(decorticate) | 2 Incomprehensible sounds | 1 No response |
| 2 Extension to pain(Decerebrate) | 1 No response |  |
| 1 No response |  |  |

**Richmond Agitation-Sedation Scale (RASS)**


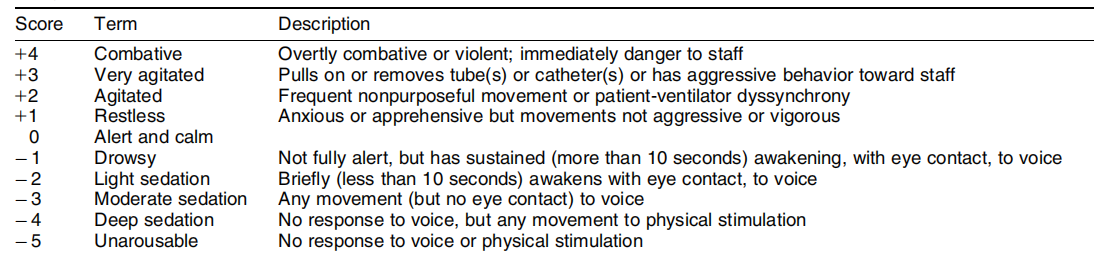


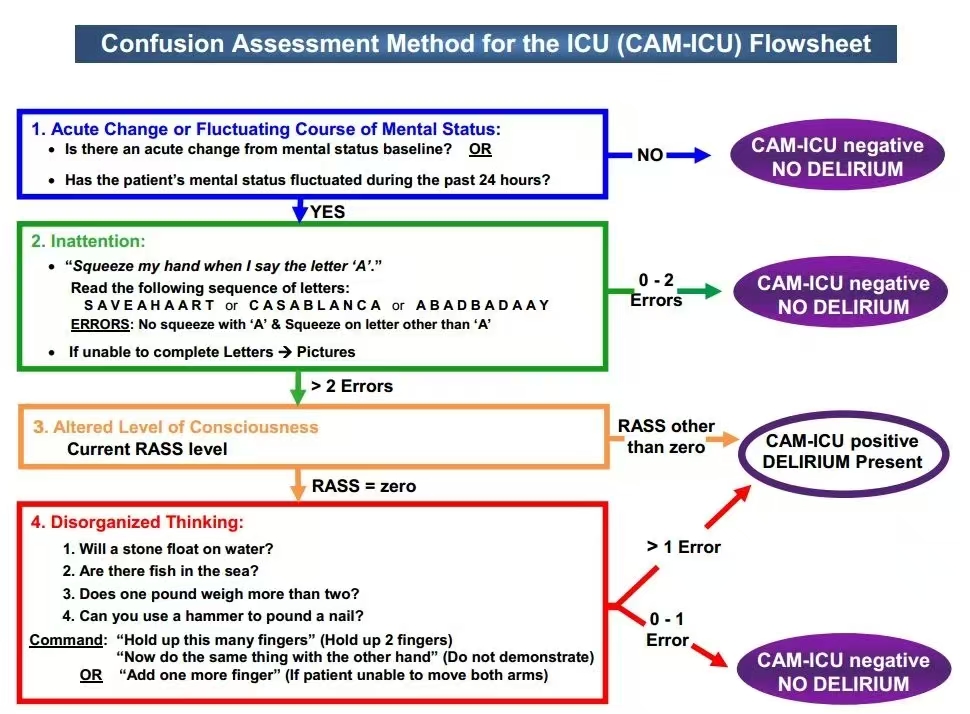

Supplement: Supplementary file 1 [file Table_1.docx]
